# Semiautomatic Volumetry of Low Attenuation of Thoracic Aortic Plaques on Curved Planar Reformations Using MDCT Angiographic Data with 0.5 mm Collimation

**DOI:** 10.1155/2018/3563817

**Published:** 2018-05-22

**Authors:** Kenji Mizutani, Izumi Torimoto, Zenjiro Sekikawa, Toshiaki Nishii, Takashi Kawasaki, Keiichiro Kasama, Takahisa Goto, Shigeo Takebayashi

**Affiliations:** ^1^Department of Anesthesiology, Yokohama City University Medical Center, Yokohama, Japan; ^2^Department of Diagnostic Radiology, Yokohama City University Medical Center, Yokohama, Japan; ^3^Department of Neurosurgery, Yokohama City University Medical Center, Yokohama, Japan; ^4^Department of Cardiovascular Surgery, Yokohama City University Medical Center, Yokohama, Japan

## Abstract

To evaluate the relationship of aortic low attenuation plaque volume (LAPV) on multidetector computed tomography (MDCT) with the abdominal aortic aneurysm (AAA), the coronary arterial disease (CAD, ≥50% stenosis), severe (≥90% stenosis) CAD, hypertension, and long-term (≥10 years) hypertension. Curved planar reformations (CPR) of three segments (the ascending, the arch, and the upper descending aorta) of the thoracic aorta were generated with attenuation-dependent color codes to measure LAPV with 0~29 HU and total noncalcified plaque volume (TNPV) with 0~150 HU in 95 patients. Correlation coefficients were employed to assess the impact of each LAPV and TNPV on AAA, CAD, severe CAD, hypertension, and long-term hypertension. Each Mean LAPV/cm and TNPV/cm was statistically greater in the aortic arch than the ascending (*p* < 0.001 on each) or the proximal descending segment (*p* < 0.001 on each). LAPV in the aortic arch has moderate correlations with AAA, severe CAD, and long-term hypertension (*r* = 0.643, 0.639, 0.662, resp.). Plaque volumes in each aortic segment can be measured clinically and the increasing LAPV in the arch may be a significant factor associated with the development of severe atherosclerosis underlying AAA, severe CAD, and long-term hypertension.

## 1. Introduction

Atherosclerosis is a degenerative disease that occurs in relatively large arteries by progressive thickening of the intima of the vessel wall [1, 2]. Accumulation of low-density lipoproteins within the intima and migration of macrophages into subendothelial spaces are regarded as the two important steps that lead to the initiation of atherosclerosis [3, 4]. Several reports in the literature have shown that atherosclerotic lesions of the thoracic aorta are a stronger predictor of coronary artery disease than conventional risk factors, and transesophageal echocardiography (TEE) has become a commonly used technique to evaluate thoracic aortic atherosclerotic plaques [5–7]. Noncalcified plaques are divided into lipid-laden plaques (soft plaques) which are hypoechoic and fibrous plaque (hard plaques) which are hyperechoic. With TEE, however, air in the trachea and left bronchus limits visualization of the upper part of the ascending aorta and the proximal arch [8]. Chatzikonstantinou et al. [8] reported that 64-row MDCT angiography could detect more plaques in a grading system throughout the aortic arch compared to TEE. Volumetric data acquired with a thinner collimation is expected to provide more accurate volume measurement [9]. Curved planar reformations (CPR) which allow a single two-dimensional image display of structures that run through multiple oblique planes have been reported to be useful in evaluating the regional calcified plaque condition of the coronary artery [10].

In this retrospective study, we evaluated the validity of semiautomatic volumetry of the low attenuation portion of the thoracic aortic plaques based on CT attenuation on CPR using MDCT angiographic data with 0.5 mm collimation. Next, we evaluated the low attenuation in the thoracic aortic plaques correlated with abdominal aortic aneurysm (AAA), coronary arterial disease (CAD), and hypertension.

## 2. Material and Methods

### 2.1. Study Group

Approval for this retrospective study was obtained from our institutional review board, and informed consent was waived. The clinical and radiological database sets at our hospital were searched for 115 consecutive patients for whom both MDCT thoracic and abdominal angiography and MDCT coronary angiography were conducted between March 2015 and June 2017. Of the 115 patients, 20 patients were excluded from this study because MDCT thoracic-abdominal angiography was performed to evaluate both AAA and the Adamkiewicz arteries using an intravenous injection of a large amount of contrast material (>500 mgI/kg). Finally, the population of this study consisted of 95 patients between the ages of 43 and 87 years. On reviewing their clinical charts, we collected data for age, gender, hyperlipidemia (treatment or high fasting cholesterol levels), diabetes (treatment or high level of HbA1c), hypertension (treatment or hypertensive blood pressure), and long-term (≥10 years) hypertension. A cardiovascular surgeon (K.K.), with 8 years of experience diagnosed AAA when MDCT showed a focal dilated aorta: an infrarenal aorta with a maximum diameter ≥ 3.0 cm or suprarenal aorta, ≥3.5 cm, on the MDCT images. CAD was diagnosed on the basis of the criteria that the MDCT coronary angiography showed ≥1 major branch had stenosis of ≥50%. In addition, severe CAD was diagnosed when the MDCT coronary angiography showed ≥1 major branch with ≥90% stenosis.

### 2.2. Volumetric Data Acquisition in MDCT Thoracic-Abdominal Angiography

The volumetric data with 0.5 mm thickness were acquired by using a 160-row or 320-row MDCT unit (Aquilion Premium or ONE; Toshiba Medical Systems, Otawara, Japan) after an intravenous bolus injection of 420–480 mgI/kg contrast material (300 or 370 mgI iopamidol) followed by 20 mL of saline at a rate of 2.5–3.5 mL/second. The data from the neck to the pubic symphysis were acquired by a 160-row scan (160 mm × 0.5 mm collimation, a beam pitch of 0.870, a rotation speed of 0.5 second, a table speed of 139 mm per rotation, and voltage of 120 kVp). Bolus tracking was used in the aorta at the level of the celiac axis for the angiographic phase. The scan of both thorax and abdomen was initiated 7 seconds after a threshold enhancement of 230 HU on the 160-row scan. The reconstructed data were of 0.5 mm thickness and 0.5 mm intervals in the angiographic phase. Postprocessing system

The postprocessing software that we used was a commercially available application, Aortic CPR Analyzer (Synapse Vincent [Synapse 3D], Fuji-Film Medical Co., Tokyo, Japan). The software was retrospectively performed in a picture archiving and communication system (PACS, Synapse, ver. 3.1, Fuji-Film Medical Co.).

**Figure 1:**
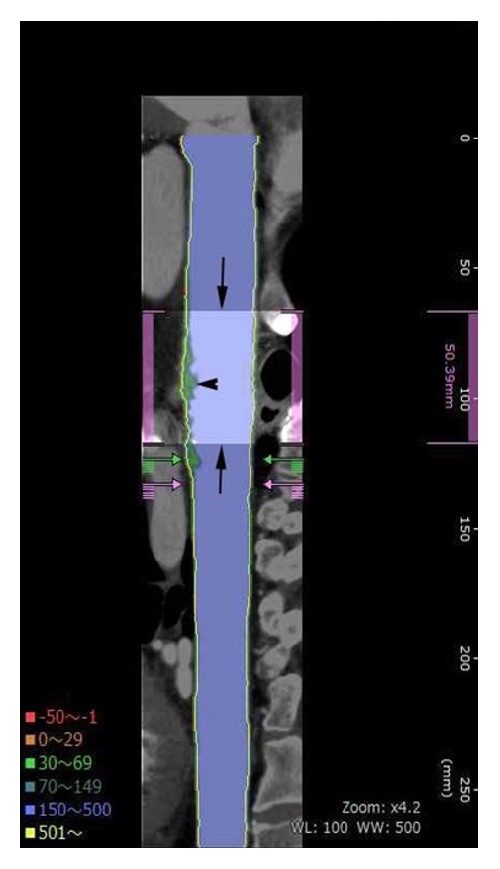
Straight CPR shows a manual segmentation of the aortic arch (arrows, light blue), 5 cm in length. Arrowhead indicates a plaque.

**Figure 2:**
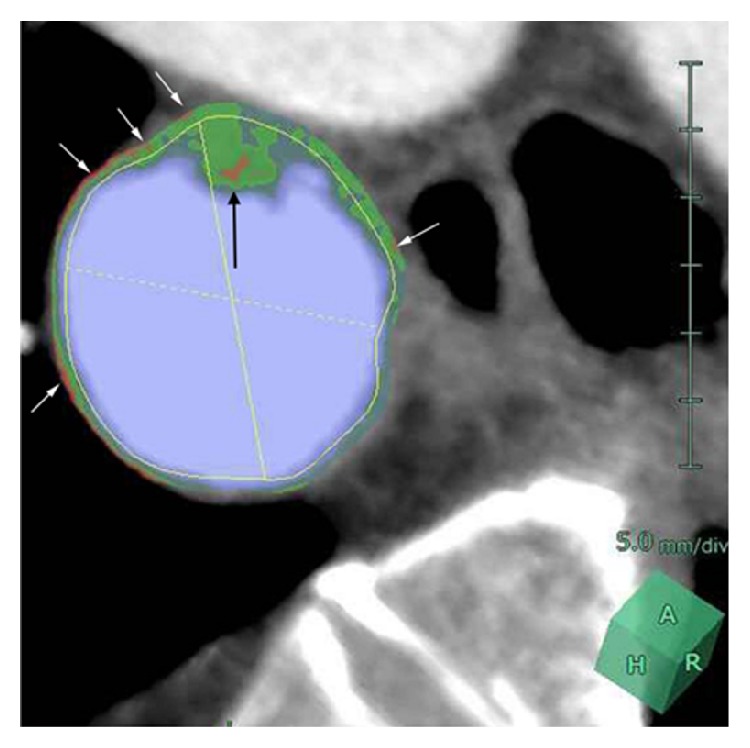
Cross-section MPR image of the descending thoracic aorta in a 70-year-old man with AAA, severe CAD, and long-term hypertension. A 6 mm thick plaque (black arrow) with mixed codes of the light green (30~69 HU), dark green (70~150 HU), and orange (0~29 HU) inside the circle indicating the normal interior of the aorta. Note the orange code indication of the low attenuation in the wall (white arrows).

**Figure 3:**
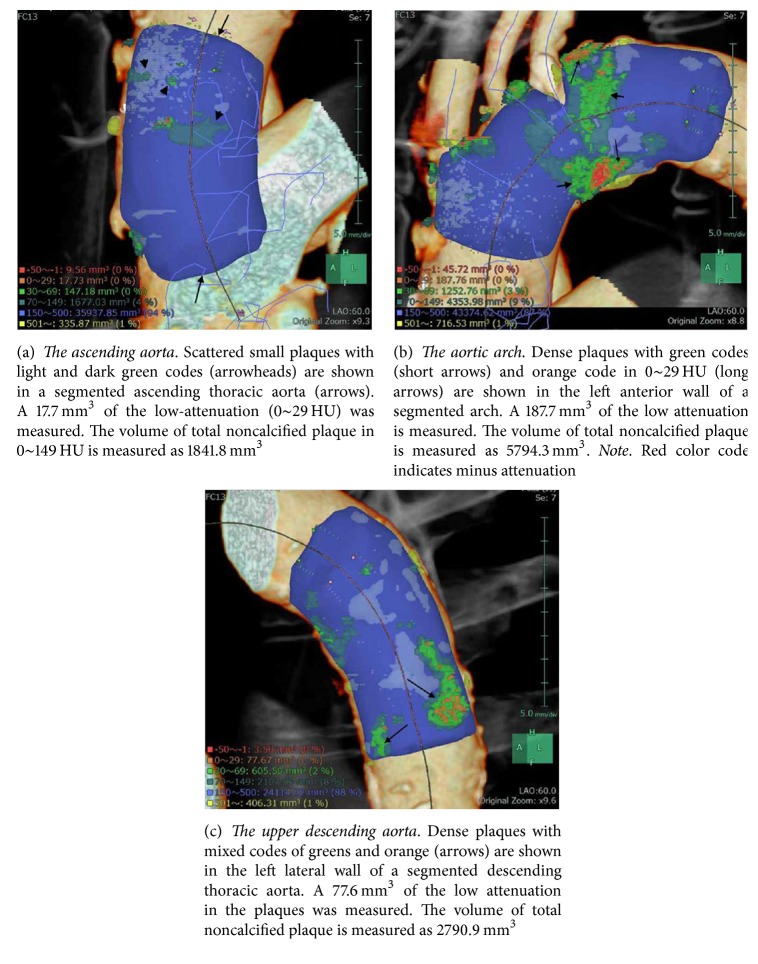
Volume rendering images of thoracic aorta in a 67-year-old man with AAA and long-term hypertension. Predetermined attenuation ranges: 0~29 HU in orange (low attenuation), 30~69 HU in light green, 70~150 HU in dark green, 151~500 HU in light blue (mixture of blood and contrast material), and 501 HU~ in yellow (calcification).

Automated creation of the aortic images included 3D volume rendering images, both straight and stretch CPR images (Figure 1), as well as the cross-section multiplanar reconstruction (MPR) images at 3 mm increment. The possible normal interior contours that were parallel to the outer wall contours were automatically displayed in the CPR and cross-section MPR images (Figure 2). The software also allowed automated quantitative measurements of discriminative color codes based on CT attenuation in the normal interior insides with predetermined attenuation ranges in Hounsfield units (HU): −50~−1 HU, 0~29 HU (low attenuation), 30~69 HU, 70~150 HU, 151~500 HU, and 501 HU ~ in yellow (calcification) [11]. Automated measurement of volumes of those attenuation ranges inside the circle indicating the normal interior of the aorta were shown on the volume rendering image (Figure 3) after the observer manually selected any aortic segment on CPR image with a reference of the volume rendering image.

### 2.3. Assessment of the Thoracic Aortic Plaques

One of the authors (KM) generated aortic CRP on the PACS and the thoracic aortic image was magnified to hid the images of the abdominal aorta because all of the users for the assessment of thoracic aortic plaques were blinded to the subjects' histories including the presence of AAA and outcomes during their independent quantitative analysis and technique performance. The thoracic aorta was classified into three segments: the ascending aorta; caudal to the brachiocephalic arterial orifice, the aortic arch; from the brachiocephalic arterial orifice to the horizontal plane distal to the orifice of the left subclavian artery, the upper descending aorta; 5 cm length caudal to the distal arch.

Morphologic assessment of the thoracic aortic thrombus in the prepared CPR of the aorta excluding the abdominal aorta was performed by two radiologists (SZ, TN) who had 15 and 12 years of experience, respectively, with vascular MDCT imaging. They reviewed multiple cross-section MPR images providing color code based CT attenuation. By consensus, they depicted the largest plaque in each thoracic aortic segment and measured its thickness with the electric caliper provided in CPR. They also noted that with the low attenuation code or ≥4 mm thickness among the largest plaques (Figure 2).

User 1 (ST), who was a radiologist with 21 years of MDCT imaging experience, manually selected each aortic segment on CPR image with a reference of the volume rendering image. The user noted the volume of low attenuation plaque with 0~29 HU and that of total noncalcified plaque with 0~150 HU in 285 aortic segments in all 95 patients. User 2 (IT), who was a technician with 4 years of experience in measurements using MDCT volumetric data, measured plaque volumes in 150 segments in a random group of 50 patients for interobserver variability. Furthermore, user 1 also repeated the measurement of each plaque in the 50 patients after a 2-month interval to assess intraobserver variability. The automated creation of aortic CPR was acquired for 5 minutes, one segmentation of the thoracic aorta using stretch CPR by manual technique was performed for 2 minutes, and the evaluation for the largest plaque in cross-section MPR of one aortic segment was performed for 5 minutes.

### 2.4. Statistical Analysis

Statistical analyses were performed using Excel add-on software, XLSTAT (Addinsoft, Cologne, Germany). Regarding the demographic factors, the categorical variables were compared with Pearson's chi-square test among patients group with AAA, CAD, severe CAD, hypertension, and long-term hypertension. Inter- and intraobserver agreements in the LAPV were measured in 150 aortic segments of the 50 patients and tested with the Bland and Altman plot method to estimate the validation of the semiautomatic measurement. Spearman's rank correlation coefficient was used to assess the correlation of AAA, CAD, severe CAD, hypertension, or long-term hypertension with each morphologic factor of the largest plaque as well as TPV and LAPV. The absolute value of the correlation coefficient was interpreted as no relationship (<0.2), weak relationship (0.2 to <0.5), moderate relationship (0.5 to <0.8), and strong relationship (0.8 to <1) [12]. Values of *p* < 0.05 were considered statistically significant.

## 3. Results

**Figure 4:**
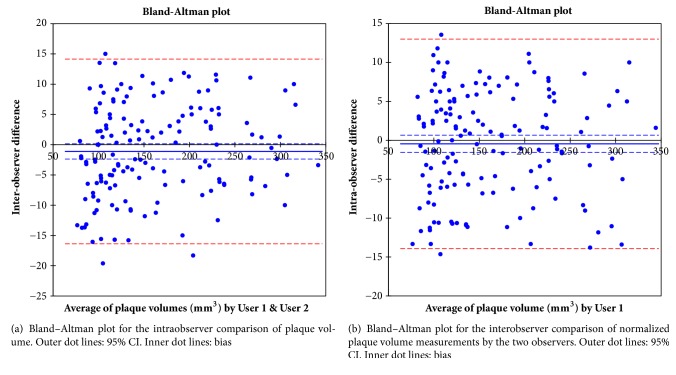
Bland–Altman plot for volumetry of the thoracic aortic plaque.

Of the 95 patients, 40 (42.1%) had AAA, 55 (57.8%) had CAD, and 60 (63.2%) had hypertension. Each severe CAD and long-term hypertension was observed in 16 patients (29.1%) and 25 patients (41.7%), respectively. There were no significant differences in the demographic factors among those patients groups (Table 1). Results of morphologic assessment of plaques on cross-section thoracic aorta MPR are shown in Table 2. The arch segment had statistically greater mean thickness and higher frequency of ≥4 mm thickness plaques than the ascending (*p*: <0.001, 0.011, resp.) and the upper descending segment (*p*: 0.002, 0.021, resp.). The frequency of the largest plaques with the low attenuation was also statistically higher in the aortic arch than the ascending (*p* < 0.001) or the upper descending segment (*p* < 0.001). A moderate positive correlation was found between the plaque thickness in the aortic arch and long-term hypertension (*r* = 0.523, *p* < 0.001).  Table 3 shows the results of the volume measurements of the thoracic aortic plaques. Each Mean low attenuation and total non-calcified plaque volume was statistically greater in the aortic arch than the ascending (*p* < 0.001 on each) or the upper descending segment (*p* < 0.001 on each). Each coefficient of AAA, severe CAD, and long-term hypertension was moderate for the low attenuation plaque volume in the arch (*r* = 0.643, 0.639, 0.662, resp.). Bland–Altman analysis indicated agreement between the two measurements of the plaque volume for intraobserver variation (*r* = 0.994, bias; −0.450 ± 6.872, 95% CI: −1.559, 0.659, *p* = 0.424, Figure 4(a)) and interobserver variation (*r* = 0.993, bias; −1.111 ± 7.786, 95% CI: −2.368, 0.145, *p* = 0.082, Figure 4(b)). This study has also demonstrated that CAD with >90% stenosis had correlation with LAPV in the aortic arch but there is no correlation between CAD with >50% stenosis and LAPV in each thoracic aortic segments.

## 4. Discussion

The importance of developing a noninvasive imaging method to assess atherosclerosis and vulnerable plaques is highly clinically relevant. One of the most important features of the vulnerable plaque is the lipid core, especially a thin-capped fibroatheroma that has a risk for disruption causing inflammation [13]. The low attenuation values 0~29 HU inside the aortic lumen on CPR or MPR using MDCT angiographic data might reflect a large lipid core in most of plaques although necrosis or hematoma might be the low attenuation. Histological findings of early atherosclerosis include an extracellular lipid pool in the outer intima of diffuse intimal thickening, which may correspond with the low attenuation area in the aortic wall. The fibrous area of the plaque is enhanced after intravenous administration of the contrast material because neovascularity has occurred by communication with the adventitial vasa vasorum [14]. The relationship between microvessels, inflammation, and lipid-core expansion in advanced atherosclerosis is also involved with intraplaque [15]. However, thoracic MDCT angiography cannot discriminate a fibroatheroma with both fibrous cap and lipid core from an atheroma with a lipid core because it shows no absolute ranges of intermediate or high attenuation that relate to the fibrous cap. Additionally, the evaluation of calcified plaque using MDCT angiographic data is difficult because there is a substantial overlap of attenuation values between calcified plaque and high attenuation blood in the lumen. Magnetic resonance imaging allows for the characterization of coronary arterial plaque or AAA composition including a thin-capped fibroatheroma and the discrimination of a lipid core [16, 17]. With regard to thoracic aortic plaque, however, the image may be degraded by artifacts due to respiratory motion and pulsatile changes due to the blood flow [17], whereas thoracic aorta CPR or MPR using MDCT angiographic data with 0.5 mm collimation is superior in spatial resolution to magnetic resonance imaging or TEE.

A recent study demonstrated that MDCT coronary angiography can provide a discriminative lipid core with low attenuation <30 HU, and this is one of the significant factors associated with thin-cap fibroatheroma on optical coherence tomography [11]. We have applied thoracic aorta CPR using the MDCT angiographic data in semiautomatic volume measurement of low attenuation (0~29 HU) in atherosclerotic plaque, and we have found an increase in LAPV in the arch which are correlated with AAA, severe CAD, and long-term hypertension, which causes further thickness of aortic wall with an apparent increase in connective tissue [18]. The aortic arch and the abdominal aorta, both which have an acute bend and large diameter side branches, are commonly found preferred sites of atherogenesis because the aorta near the branch orifices has regions of disturbed flow causing low wall-shear stresses and the localization of concentrated low-density lipoproteins [19]. Liu et al. [3] reported that severe low-density lipoprotein concentration can be predicted in the exit of the aortic arch and the lower descending thoracic aorta with taper. A recent study using TEE showed more plaques with ≥4 mm thickness in the descending aorta than the aortic arch, and the large plaque in the descending thoracic aorta had the strongest independent association with the presence of significant CAD with >70% stenosis [7]. In this study using MDCT angiographic data, however, the morphology and volumes of plaques in the upper descending thoracic aorta have no correlation with CAD or severe CAD with ≥90% stenosis. The disagreement between the two techniques can probably explained as follows: (1) There remains a risk for underestimation of the aortic arch plaques in TEE because the proximal part of the aortic arch is often poorly visualized in TEE due to the juxtaposition of the trachea and the right main bronchus between the esophagus and the aorta [8]. In fact, MDCT angiography was reported to identify more plaques throughout the aortic arch compared to TEE [8]. (2) In the TEE study, the evaluation for the plaques was performed in the whole descending thoracic aorta including the preferred site of atherogenesis. (3) The plaques in the arch exit which is categorized into the arch plaques in this study might be classified into the descending aortic plaques.

There are some limitations to this study. First, in this retrospective study, we included quantitative evaluation of the measurements, and the observer may have affected the measurements of the MDCT images. Second, there is no histological confirmation of the low attenuation plaque. Third, this study has included selected high-risk populations.

In conclusion, the volume of the aortic plaque can be measured clinically by thoracic aorta CPR using the MDCT angiographic data. The technique may be used to provide both quantitative and qualitative information of the vulnerable plaque which is characterized by CT attenuation in atherosclerosis-related disorders.

## Figures and Tables

**Table 1:** Demographics in 95 subjects correlated with AAA, CAD, and hypertension.

		AAA	CAD >50% stenosis	CAD >90% stenosis	Hypertension	Hypertension >10 years	*p* values
Age [years] (mean ± sd)	69.2 ± 10.9	72.4 ± 9.1	70.1 ± 11.0	71.6 ± 9.1	69.3 ± 12.5	71.6 ± 9.9	NP
Male/female	83/12	33/7	18/3	13/3	51/9	21/4	0.993
Hyperlipidemia no. (%)	70 (73.7)	33	44	11	45	21	0.691
Diabetes no. (%)	33 (31.2)	12	21	7	22	9	0.885

	Total	40 (42.1)	55 (57.8)	16 (16.8)	60 (63.2)	25 (26.3)	

AAA: abdominal aortic aneurysm, CAD: coronary arterial disease, sd: standard deviation, NP: not performed.

**Table 2:** Characteristics of noncalcified largest plaques on cross-section thoracic aorta multiplanar reformation and univariate correlations with AAA, CAD, and hypertension.

Noncalcified largest plaque mean ± sd/No. (%)	AAA	CAD ≥50% stenosis	Severe CAD ≥90% stenosis	Hypertension	Long-term (≥10 years) hypertension
*The ascending aorta*					
Thickness 1.60 mm ± 1.12	0.120 (0.247)	0.248 (0.015)	0.153 (0.138)	−0.110 (0.288)	0.227 (0.027)
≥4 mm thickness 9 (9.5)	0.307 (0.003)	0.264 (0.010)	0.130 (0.208)	0.133 (0.199)	0.029 (0.782)
With low attenuation 18 (18.9)	0.023 (0825)	0.154 (0.137)	−0.003 (0.979)	0.042 (0.684)	−0.099 (0.340)
*The aortic arch*					
Thickness 2.88 mm ± 1.18	0.126 (0.225)	0.006 (0.703)	0.441 (<0.001)	0.213 (0.039)	0.512^*∗*^ (<0.001)
≥4 mm thickness 23 (24.2)	0.115 (0.265)	0.060 (0.562)	0.450 (<0.001)	0.143 (0.167)	0.423 (<0.001)
With low attenuation 46 (48.4)	0.155 (0.134)	0.155 (0.134)	0.186 (0.071)	−0.012 (0.911)	0.313 (0.002)
*The upper descending aorta*					
Thickness 2.52 mm ± 1.04	0.267 (0.009)	0.296 (0.004)	0.171 (0.098)	−0.020 (0.849)	0.239 (0.009)
≥4 mm thickness 10 (10.5)	0.194 (0.060)	0.140 (0.176)	0.191 (0.069)	−0.006 (0.953)	0.266 (0.635)
With low attenuation 17 (17.9)	0.158 (0.126)	0.067 (0.517)	0.031 (0.762)	−0.066 (0.522)	0.027 (0.793)

AAA: abdominal aortic aneurysm, CAD: coronary arterial disease, ^*∗*^moderate correlation.

**Table 3:** Univariate correlations of each low attenuation and total noncalcified plaque volume on curved planar reformation with AAA, CAD, and hypertension.

Volumes (mm^3^) of noncalcified largest plaque mean ± sd	AAA	CAD (≥50% stenosis)	Severe CAD (≥90% stenosis)	Hypertension	Long-term (≥10 years) hypertension
*Ascending aorta*					
Low attenuation 55.9 ± 63.8	−0.030 (0.774)	0.009 (0.934)	0.099 (0.339)	−0.117 (0.260)	0.204 (0.047)
Total 2016.6 ± 985.3	−0.039 (0.707)	−0.033 (0.749)	−0.117 (0.259)	−0.051 (0.620)	0.040 (0.699)
*Aortic arch*					
Low attenuation 180.3 ± 115.9	0.643^*∗*^ (<0.001)	0.319 (0.002)	0.639^*∗*^ (<0.001)	0.332 (0.001)	0.662^*∗*^ (<0.001)
Total 5515.0 ± 2121.4	0.434 (<0.001)	0.120 (0.247)	0.428 (<0.001)	0.241 (0.019)	0.351 (0.001)
*Upper descending aorta*					
Low attenuation 37.6 ± 39.8	0.117 (0.339)	0.046 (0.657)	0.213 (0.038)	0.010 (0.921)	0.132 (0.201)
Total 2187.5 ± 1104.5	0.146 (0.159)	0.023 (0.822)	0.168 (0.102)	0.109 (0.293)	0.092 (0.372)

AAA: abdominal aortic aneurysm, CAD: coronary arterial disease; sd: standard deviation,  ^*∗*^moderate correlation.

## Abbreviations

LAPV: Low attenuation plaque volume
TNPV: Total noncalcified plaque volume
MDCT: Multidetector computed tomography
AAA: Abdominal aortic aneurysm
CAD: Coronary arterial disease
TEE: Transesophageal echocardiography
CPR: Curved planar reformations
PACS: Picture archiving and communication system
MPR: Cross-section multiplanar reconstruction.
